# Functionalization of Mesoporous Semiconductor Metal Oxides for Gas Sensing: Recent Advances and Emerging Challenges

**DOI:** 10.1002/advs.202204810

**Published:** 2022-11-14

**Authors:** Xuanyu Yang, Yu Deng, Haitao Yang, Yaozu Liao, Xiaowei Cheng, Yidong Zou, Limin Wu, Yonghui Deng

**Affiliations:** ^1^ Department of Chemistry Department of Gastroenterology and Hepatology Zhongshan Hospital Zhangjiang Fudan International Innovation Center State Key Laboratory of Molecular Engineering of Polymers Shanghai Key Laboratory of Molecular Catalysis and Innovative Materials iCHEM Fudan University Shanghai 200433 China; ^2^ State Key Laboratory for Modification of Chemical Fibers and Polymer Materials College of Materials Science and Engineering Institute of Functional Materials Donghua University Shanghai 201620 China; ^3^ School of Materials Science and Engineering Nanchang Hangkong University Nanchang 330063 China; ^4^ Institute of Energy and Materials Chemistry Inner Mongolia University Hohhot 010021 China

**Keywords:** gas sensor, interface catalysis, mesoporous materials, metal oxide, semiconductor

## Abstract

With the emerging of the Internet of Things, chemiresistive gas sensors have been extensively applied in industrial production, food safety, medical diagnosis, and environment detection, etc. Considerable efforts have been devoted to improving the gas‐sensing performance through tailoring the structure, functions, defects and electrical conductivity of sensitive materials. Among the numerous sensitive materials, mesoporous semiconductor metal oxides possess unparalleled properties, including tunable pore size, high specific surface area, abundant metal–oxygen bonds, and rapid mass transfer/diffusion behavior (Knudsen diffusion), which have been regarded as the most potential sensitive materials. Herein, the synthesis strategies for mesoporous metal oxides are overviewed, the classical functionalization techniques of sensitive materials are also systemically summarized as a highlight, including construction of mesoporous structure, regulation of micro‐nano structure (i.e., heterojunctions), noble metal sensitization (e.g., Au, Pt, Ag, Pd) and heteroatomic doping (e.g., C, N, Si, S). In addition, the structure–function relationship of sensitive materials has been discussed at molecular‐atomic level, especially for the chemical sensitization effect, elucidating the interface adsorption/catalytic mechanism. Moreover, the challenges and perspectives are proposed, which will open a new door for the development of intelligent gas sensor in various applications.

## Graphical abstract

The intelligent information age has brought new development opportunities for gas sensors. Herein, a brand‐new perspective is proposed to design functional mesoporous metal oxides for sensing performance optimization. The structure–function relationship of sensitive materials is discussed in molecular‐atomic level, and the challenges and perspectives are proposed for the rationally design of advanced mesoporous semiconductor sensing materials for intelligent sensors.

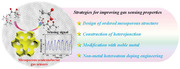

## 1 Introduction

**Figure 1:**
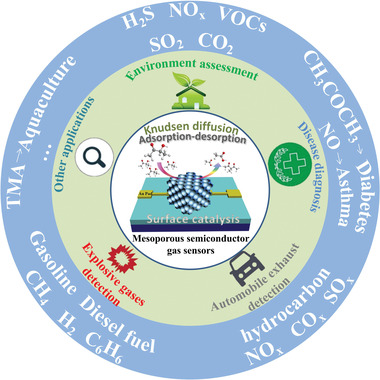
The broad applications of functional gas sensors based on semiconductor metal oxides.

Recently, sensing technologies have received numerous concerns with the booming development of the Internet of Things, Industrial Internet of Things (IIoT) and Internet‐connected automation, due to their reliable monitoring and flexible signal transmission.^[^ ^1^ ^]^ As an important branch of sensing technology, chemiresistive gas sensor plays an irreplaceable role in various applications (**Figure** **1**), such as environment assessment, disease diagnosis, automobile exhaust detection, and explosive gas monitoring.^[^ ^2^ ^]^ Sensitive materials are the core components of gas sensors, and various nanomaterials (porous and non‐porous) with diverse structures (e.g., size, morphology, crystal form, crystallinity) and components (e.g., metal oxides, noble metal, graphene, polymers) have been exploited to detect different targeted gases (e.g., CO, NO*_x_*, H_2_S, NH_3_, acetone, ethanol, CH_4_, H_2_). Among these gas sensors, semiconducting metal oxides (MOS) based gas sensors have gained considerable attention in multidisciplinary applications owing to their high sensitivity, fast response, low‐cost, and excellent long‐term stability.^[^ ^3^ ^]^ However, low operating temperature, excellent selectivity, and trace gas detection (i.e., low detection limit) are essential to the practical application of MOS‐based gas sensors, and it still remains a great challenge to break these performance bottlenecks, and thus it is vital to exploit new materials for the next‐generation smart gas sensors.

Over the past few decades, significant efforts have been devoted to improve the gas‐sensing performance by rationally designing sensitive materials, including porous structure, heterojunctions, energy band structure, and heteroatomic doping.^[^ ^4^ ^]^ Inspired by the space charge layer model, potential barrier on grain boundary model and the gas diffusion model, the MOS materials with high specific surface area, small grain size (less than double thickness of Debye length), rich porosity, surface active sites, and oxygen species (i.e., O^2−^, O_2_^−^ and O^−^) are highly desired in the high‐performance gas sensor. From this perspective, mesoporous materials have been recently considered as worthwhile candidates to resolve the above problems owing to their interconnected channels, large pore size, high specific surface area and abundant surface active sites. Such a feature is highly desired for the adsorption–desorption process, gas diffusion, and surface‐catalyzed reaction involved in sensing behavior, leading to the fast response and recovery dynamics, as well as the superior sensitivity.^[^ ^5^ ^]^ Moreover, the gas diffusion is enhanced in the mesopores and is dominant with Knudsen diffusion. In this regard, the gas molecules can freely diffuse within mesoporous matrix and react with internal sites, which can significantly improve the sensing performance.

Owing to the outstanding merits of mesoporous semiconducting metal oxides, various methods have been employed to fabricate the MOS‐based sensors in the past decade. In general, these mesoporous sensitive materials are mainly synthesized via classical template technology (i.e., soft‐template and hard‐template). Particularly, the template method offers a platform for the regulation of interfacial sites and superficial structures, which can optimize and remodel their surface chemical environments (e.g., acidity, functional groups, and valence state) and the electronic properties (e.g., energy band structure, electron conductivity).^[^ ^6^ ^]^ Therefore, revealing the structure‐function relationship as well as developing general sensitization mechanisms for designing porous MOS gas sensing materials is an urgent demand and scientific problem. Although there are already some reviews on the topic of MOS‐based gas sensing materials, the rational design of mesoporous structure for sensing applications and the fine‐tuning of surface/interface properties, as well as the elucidation of the relevant gas‐sensing enhancement mechanisms have been rarely discussed.

Herein, recent research progresses on mesoporous MOS‐based gas sensors were reviewed systematically. The synthesis methods of mesoporous MOS materials based on the template approach and free‐template method are summarized, and endless functional mesoporous MOS have been synthesized with unique physicochemical properties, including high specific surface area, tunable pore structure (e.g., pore size, pore volume, pore wall) and surface structure (e.g., morphology, roughness, charge). As a research highlight, the efficient sensitization strategies of gas sensors, including the construction of heterojunctions, incorporation with noble metal (e.g., Au, Pt, Pd) and doping with alternative heteroatoms (e.g., C, N, S and Si), and structure–function relationship for gas sensor have been discussed and compared systematically. Additionally, the microscopic properties of hybrid materials (e.g., carrier transport, energy bands, structural defects and recombination) and the mechanism of gas‐solid chemical reactions between gases and active interfaces are also discussed in depth. Finally, the challenge and the perspective are proposed with an aim to guide rational design of advanced porous MOS materials.

## 2 Strategies to Enhance Sensing Performance

### 2.1 Construction of Mesoporous Metal Oxides

Mesoporous metal oxide semiconductors with tunable pore structure and precisely controlled surface properties have attracted extensive research interests in gas sensing fields owing to their high specific surface area, adjustable framework compositions, and unique electronic structures. Particularly, Knudsen diffusion occurs when the scale of the diffusion system is smaller than the mean free path of the particles.^[^ ^7^, ^8^ ^]^ As defined in Equation 1, where *L* represents physical length scale, i.e., pore size.

(1) $$K_{n}=\frac{\bar{\lambda }}{L}$$

Higher Knudsen value indicates larger mean free path of fluid than physical length scale, thus facilitating fluid to pass more freely through the representative container. The diffusion coefficient of Knudsen diffusion *D*_k_ is defined in Equation 2, in which *r* stands for pore size, *R* stands for universal gas constant and *M* stands for molecular weight of the particles. *D*_k_ value is proportional to the square root of the reciprocal of molecular weight *M*, and thus gas molecules with smaller molecular weight can diffuse faster than those with larger ones. This enables Knudsen diffusion to have a slightly higher selectivity than bulk Poiseuille flow, but still not sufficient in practical application.

(2) $$D_{k}=\frac{4r}{3}\sqrt{\frac{2\mathrm{RT}}{\pi M}}$$

Particularly, when the value of *D*_k_ is comparable with pore sizes (1–100 nm), Knudsen diffusion becomes dominant, and larger pores can accelerate gas molecules to diffuse freely, while the confined diffusion is more significant in smaller pores.^[^ ^1a^ ^]^ As a result, the specific designed ordered mesoporous structure (pore size: 2–50 nm) is extremely desired for the gas sensing process.

#### 2.1.1 Hard‐Templating Method

**Figure 2:**
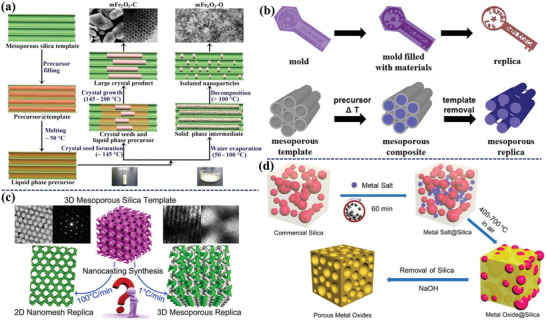
a) The container effect in the nanocasting synthesized mesoporous Fe_2_O_3_ with tunable size. Adapted with permission.^[^ ^9^ ^]^ Copyright 2011, American Chemical Society. b) The synthesis route of mold or nanocasting based on the hard template of ordered mesoporous materials. Adapted with permission.^[^ ^11^ ^]^ Copyright 2017, American Chemical Society. c) The synthesis process of mesoporous replica based on 3D mesoporous silica template. Adapted with permission.^[^ ^15^ ^]^ Copyright 2019, American Chemical Society. d) Classical mechanochemical nanocasting technique to synthesize porous metal oxide. Adapted with permission.^[^ ^16^ ^]^ Copyright 2018, American Chemical Society.

With the widespread application of mesoporous materials, the synthesis methods of mesoporous materials have been greatly developed, such as the hard‐templating approach (i.e., nanocasting) and soft‐templating approach, especially for the design of ordered mesoporous MOS. The hard‐templating method usually relies on the utilization of pre‐synthesized mesoporous materials (e.g., mesoporous silica like SBA‐15, mesoporous carbon like CMK‐3), and the guest precursors are impregnated into the porous channel to fill the mesopores, followed by thermal treatment to convert the inorganic metallic precursor into crystalline metal oxides and the porous crystalline metal oxides can be obtained by removal of the sacrificial template (**Figure** **2**a).^[^ ^9^ ^]^ In general, the hard‐templating method has the following advantages: 1) without structure directing agents, and the complex hydrolysis‐condensation process of inorganic precursor can be also ignored. Therefore, the synthesis method is universal for various mesoporous materials; 2) mesoporous silica or carbon templates are relatively stable and their pore channels can be readily filled by suitable inorganic precursors to construct various mesoporous materials, such as mesoporous metal,^[^ ^10^ ^]^ metal oxide,^[^ ^11^ ^]^ metal sulfide,^[^ ^12^ ^]^ metal nitride,^[^ ^13^ ^]^ and metal carbide.^[^ ^14^ ^]^ Typically, Tüysüz and co‐workers adopted ordered mesoporous silica (i.e., MCM‐41, KIT‐6 and SBA‐15) as hard template to successfully synthesize ordered mesoporous transition metal oxides, including Co_3_O_4_, NiO, Fe_2_O_3_ and Mn_3_O_4_ (Figure 2b).^[^ ^11^ ^]^ Although template‐assisted method is usually employed to prepare porous metal oxides, such as 3D bulk and 2D nanomesh (Figure 2c),^[^ ^15^ ^]^ this approach is commonly performed in wet conditions, which requires solvents, soluble metal salt precursors, and prolonged drying. To address those issues of the wet procedure, Xiao et al.^[^ ^16^ ^]^ developed a mechanochemical nanocasting method to synthesize various porous metal oxides including CeO_2_, Fe_2_O_3_, CuO*_x_*‐CeO*_y_*, ZrO_2_ and CuO*_x_*‐CoO*_y_*‐CeO*_z_* with high specific surface areas (Figure 2d), and the rigid scaffold of commercial porous silica (Com‐SiO_2_) was used in this synthesis process. The inorganic precursor was directly mixed with Com‐SiO_2_ by ball milling, and the inorganic precursor could be well‐dispersed within the interstitial pores of Com‐SiO_2_. Porous crystalline metal oxides were obtained by removal of Com‐SiO_2_ via NaOH etching, while the obtained metal oxides exhibited disordered pore structure and weak channel connectivity. Limited by the rigid structure of as‐prepared templates such as mesoporous silica and carbon, the pore structure (such as size, types, window, connectivity) of targeted materials is difficult to regulate, and thus this method is expensive, tedious operation and unsuitable for mass production.

#### 2.1.2 Soft‐Templating Method

By contrast, the soft‐templating approach has been regarded as one of the most efficient and flexible method to design ordered mesoporous materials via the controllable co‐assembly process. In general, the classical soft‐templating approach is mainly accomplished via solvent evaporation‐induced self‐assembly (EISA) or solvent evaporation induced aggregation assembly (EIAA), where the assembly driving forces including hydrogen bonds, electrostatic interaction and covalent bonds between inorganic precursors and organic surfactant (e.g., block copolymers) can induce the formation of uniform organic–inorganic micelles via the complex hydrolysis‐condensation behavior. The hybrid micelles can closely pack into ordered mesoporous structure with the evaporation of solvent (e.g., tetrahydrofuran (THF), acetone, methanol, etc.).^[^ ^17^ ^]^ Subsequently, the crystalline mesoporous metal oxides can be achieved after removing the surfactant through calcination. However, there are still remained several difficulties in the synthesis of ordered mesoporous metal oxides, such as the weak interaction between inorganic species and surfactant, the poor controllability of hydrolysis‐condensation process and easy collapse of porous structure during the high‐temperature treatment.^[^ ^18^ ^]^

**Figure 3:**
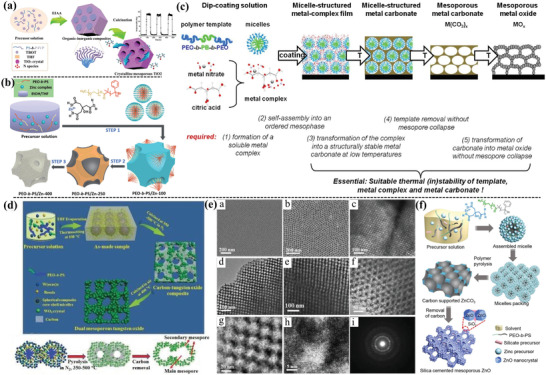
a) The typical synthesis route of the ordered mesoporous TiO_2_ based on EIAA strategy. Adapted with permission.^[^ ^19^ ^]^ Copyright 2018, Elsevier. b) Citric acid chelate assisted EISA synthesis process of crystalline mesoporous ZnO. Adapted with permission.^[^ ^22^ ^]^ Copyright 2016, The Royal Society of Chemistry. c) Synthesis and deduced conditions toward the design of mesoporous metal oxides based on metal carbonate intermediates. Adapted with permission.^[^ ^23^ ^]^ Copyright 2013, American Chemical Society. d) Resol‐assisted EISA technique for the synthesis of the ordered dual mesoporous WO_3_ and pore evolution behavior. e) FESEM and TEM images of the prepared mesoporous WO_3_. Adapted with permission.^[^ ^25^ ^]^ Copyright 2018, Wiley. f) Ligand‐chelating assisted EISA strategy to synthesize silica cemented mesoporous ZnO. Adapted with permission.^[^ ^39^ ^]^ Copyright 2019, American Chemical Society.

Particularly, the interaction between inorganic species and surfactant can be enhanced by using the appropriate precursor and organic polymer templates. For example, Yang et al.^[^ ^19^ ^]^ synthesized N‐doped ordered mesoporous TiO_2_ through using lab‐made amphiphilic diblock copolymer polystyrene‐*b*‐poly (4‐vinylpyridine) (PS‐*b*‐P4VP) as structure directing agent and titanium tetrabutoxide (TBOT) as titanium source (**Figure** **3**a). Due to the strong chelation interaction between pyridine‐N and Ti species, ordered mesoporous TiO_2_ with crystalline framework can be obtained without extra additives during synthesis. Additionally, Lunkenbein et al.^[^ ^20^ ^]^ used the diblock copolymer polybutadiene‐*b*‐poly‐(2‐dimethylaminoacrylate) (PB‐*b*‐PDMAEMA) and H_3_[PMo_12_O_40_] to construct thin films with meso‐morphology based on the strong electrostatic interaction between protonated PDMAEMA segment and PMo^3−^. Similarly, Zhang et al.^[^ ^21^ ^]^ synthesized the multilevel mesostructure by tuning the electrostatic interaction between PS‐*b*‐P2VP and H_4_SiW.

Besides, small molecule ligands can be regarded as “bridge” to enhance the interaction between inorganic species and surfactant via coordination interaction to promote the organic–inorganic assembly. For example, Zhou et al.^[^ ^22^ ^]^ synthesized mesoporous zinc oxides through adopting citric acid as assistant to improve the interaction between Zn^2+^ and PEO segments (Figure 3b), where citric acid molecules could induce the formation of partially hydrolyzed zinc citric species. It can also interact with the ethylene oxide (EO) moieties of the template molecules via hydrogen bonding (i.e., Zn^2+^—O—C—O···H^+^···EO). Similarly, Eckhardt et al.^[^ ^23^ ^]^ also used citric acid as the chelating agent to interact with the metal nitrate to form stable metal complex which further interacted with block copolymer template molecules to assemble into ordered mesophase (Figure 3c). With this assembly technique, porous ZnO and Co_3_O_4_ films with controlled meso‐porosity can be obtained. In addition, Fan et al.^[^ ^24^ ^]^ demonstrated that the controlled binding of reactive metal ions with acetic acid could generate nanometer‐sized particles for a diverse collection of inorganic materials with homogeneous condensation kinetics, in which the chelate acetate groups acted as the bridge to connect the amphiphilic block copolymers and inorganic species. It also showed that the crosslinking degree of composite gel networks was decreased due to the replacement of hydrolyzable alkoxy groups by acetate groups. Hence, the ligands including the specifically designed chelating agents can act as a bridge to connect the inorganic species and surfactant, facilitating the formation of a well‐organized mesostructure.

Notably, the fast hydrolysis‐condensation behavior of framework precursors such as metal chlorides or metal alkoxides, can cause serious phase separation and aggregation of inorganic species, and even result in a disordered mesoporous structure with irregular pore size. In order to solve the above problems, a series of regulating agents, such as hydrochloric acid and acetylacetone have been employed to control the hydrolysis rate of metal precursors. In particular, the small acid molecules not only slow down the hydrolysis of metal precursors but also interact with the surfactant via protonation to enhance the interaction between surfactant and metal precursors.^[^ ^25^ ^]^ For examples, Li et al.^[^ ^26^ ^]^ synthesized ordered mesoporous WO_3_ material with highly crystalline framework by using the hydrochloric acid as the adjusting agent and amphiphilic poly(ethylene oxide)‐*b*‐polystyrene (PEO‐*b*‐PS) diblock copolymers as the template. Hydrochloric acid helps to inhibit the hydrolysis of precursor (i.e.,WCl_6_) and meanwhile it protonates the PEO segments of PEO‐*b*‐PS, which enhances the hydrogen bonds between partially hydrolyzed hydrophilic tungsten species and the PEO segments. In addition, Wei et al.^[^ ^27^ ^]^ used nitric acid as the assisting agent to synthesize the mesoporous Al_2_O_3_ based on PEO‐*b*‐PS as soft template and aluminum acetylacetonate as precursor, and it confirmed that the nitric acid could strongly inhibit the hydrolysis of aluminum species and the acetylacetonate groups acted as the chelating agent to form the complex which further controlled the hydrolysis process. Typically, the complexing agents are explored to prevent the inorganic species aggregation. For example, Xiao et al.^[^ ^28^ ^]^ used the acetylacetone to retard the hydrolysis of SnCl_4_ and generate acetylacetone‐stabilized SnO*_x_* nanoclusters, and it showed that the tin species could be quickly hydrolyzed into tin oxide nanoclusters. However, the aggregation of tin oxide nanoclusters can be effectively suppressed in the presence of acetylacetone, and the diameter of tin oxide nanoclusters can be readily controlled for further coassembly with copolymers to form the mesostructure. Notably, the direct co‐assembly of presynthesized ultrasmall nanocrystals with block copolymers can avoid the influence of hydrolysis of metal precursors on the assembly process.^[^ ^29^ ^]^ Therefore, this method was extremely desired for the synthesis of target metal oxides whose precursors were easily hydrolyzed, and various mesoporous materials including TiO_2_, CeO_2_, In_2_O_3_, SnO_2_, CdSe, and FePt have been reported via this method.^[^ ^30^ ^]^

Conventionally, commercial Pluronic surfactants consisting of non‐ionic poly(ethylene oxide) and poly(propylene oxide), such as P123,^[^ ^31^ ^]^ F127,^[^ ^32^ ^]^ and F108^[^ ^33^ ^]^ have been widely explored to synthesize porous materials. However, it is difficult to obtain highly crystalline framework by using these templates due to their low thermal stability. As a result, they usually lead to partially crystallized porous structure or even metal oxides with collapsed structure after annealing at high temperatures.^[^ ^34^ ^]^ Therefore, the amphiphilic block copolymers with higher thermal stability and high‐molecular weights such as PS‐*b*‐P4VP,^[^ ^19^ ^]^ PS‐*b*‐P2VP,^[^ ^21^ ^]^ poly(ethylene‐co‐butylene)‐*b*‐poly(ethylene oxide) polymer (KLE),^[^ ^35^ ^]^ poly (isoprene‐block‐ethylene oxide) (PI‐*b*‐PEO)^[^ ^36^ ^]^ and PEO‐*b*‐PS,^[^ ^37^ ^]^ have been used to synthesize the highly crystallized mesoporous metal oxides with large pore size. Typically, Lee et al.^[^ ^38^ ^]^ first reported a combined soft and hard templates assembly method (CASH) based on the structure‐directing agent PI‐*b*‐PEO. The as‐made sample was firstly treated under the inert environment (e.g., N_2_ or Ar), and the PEO segment could be easily decomposed, while the PI segment could be converted to rigid sp^2^‐carbon to support the mesoporous framework. Therefore, highly crystalline mesoporous metal oxides can be obtained even under high‐temperature calcination in air. In recent years, our group further developed the CASH method by using the PEO‐*b*‐PS diblock copolymers as the templates, and various mesoporous metal oxides such as TiO_2_,^[^ ^37a^ ^]^ WO_3_,^[^ ^26^ ^]^ SnO_2_,^[^ ^28^ ^]^ Al_2_O_3_,^[^ ^27^ ^]^ ZnO^[^ ^37d^ ^]^ and Ce–Zr solid solution^[^ ^37c^ ^]^ have been successfully synthesized based on gas–liquid interface assembly. Furthermore, the doping of soluble phenolic resin (resol) and silicate species in the meso‐framework was proven to be effective in preventing the collapse of porous structure during high temperature treatment. Inspired by this perspective, Li et al.^[^ ^25^ ^]^ demonstrated a pore engineering strategy to synthesize ordered mesoporous WO_3_ with well‐connected bimodal pores and crystalline pore walls based on hydrophilic resol as the sacrificial carbon source (Figure 3d), and it could interact preferentially with PEO domains and serve as a glue to bridge the tungsten species and PEO‐*b*‐PS templates. In addition, the resol and copolymers both serve as the rigid support to prevent the structure collapse of mesostructured oxide wall during high temperature treatments and the highly crystallized hierarchical mesoporous WO_3_ can be obtained (Figure 3e). Based on the supporting effect of the rigid matrix, Zhou et al.^[^ ^39^ ^]^ reported controlled synthesis of silica‐cemented mesoporous ZnO material by using citric acid as the chelating agent to enhance the weak interaction between zinc precursor and PEO‐*b*‐PS surfactants (Figure 3f). Besides, the silicate oligomer derived from the intentionally introduced tetraethyl orthosilicate (TEOS) could interact with citric acid via hydrogen bonds to ensure co‐assembly with the zinc precursor, preventing the collapse of porous structure during the high temperature treatment. Moreover, using crystallized nanocrystals as the precursor can replace inorganic metal precursor to assemble with surfactant, which can also avoid mesostructure collapse and uncontrolled hydrolysis behavior of inorganic precursor.^[^ ^37^, ^40^ ^]^

As mentioned above, several elegant assembly strategies, including ligand‐assisted (chelating agents and adjusting agents) soft‐template strategy, sp^2^ residual‐carbon supporting strategy, and amorphous components (phenolic resin and silicate species) assisted assembly strategy, can guarantee the assembly behavior more controllable. Thus, mesoporous semiconductors with tunable pore architecture and precisely controlled surface properties can be rationally synthesized to achieve the high‐performance gas sensor.

#### 2.1.3 Self‐Templating Synthesis

It is widely recognized that metal‐organic frameworks (MOFs) are typical porous organic/inorganic hybrid materials, which can be self‐assembled from their corresponding inorganic metal ions/clusters and organic linkers. Benefit for their unique porous structure and components, most of them have exhibited great application potential in catalysis, sensors, and energy conversion. In recent years, MOFs were usually employed as the sacrificial template to construct various functional materials with high conductivity and structural stability, such as heteroatom‐doped porous carbons, metal oxides, metal (oxides)/carbon composites, and other metal‐containing nanomaterials. Particularly, porous metal oxides derived from MOFs showed great potentials in gas sensors due to their high specific surface area, tailored porosity, and easy functionalization with other heteroatoms or metal/metal oxides. Typically, ZIF‐8 was first used to load Pd (defined as Pd@ZIF‐8),^[^ ^41^ ^]^ and the obtained Pd@ZIF‐8 suspension was added to the electrospinning solution to form the as‐spun composites, which were further treated at high temperatures. The synthesized Pd@ZnO‐WO_3_ nanofibers (NFs) exhibit unparalleled toluene sensing performance. Similarly, Zhang et al.^[^ ^42a^ ^]^ reported the MOFs‐derived porous ZnO/Co_3_O_4_ nanoheterostructure by a facile precipitation method, and the materials exhibited high sensing performance towards acetone. Lu et al.^[^ ^42b^ ^]^ synthesized the core–shell structured mesoporous Sn‐doped NiO by bimetallic MOFs‐derived method, which showed excellent xylene sensing performance. It found that the MOFs derivatives endowed the materials with highly connected mesoporous structure, which contributed to the mass diffusion and fast response rate, and the sensors were highly sensitive to xylene (*R*_gas_/*R*_air_ = 46.7 to 100 ppm). Additionally, Wang et al.^[^ ^42c^ ^]^ constructed the NiO‐doped CuO composites by using CuNi‐based MOFs as the template, and the fabricated sensors exhibited excellent sensing performance toward H_2_S (106%, 50 ppb at ‐25 °C). The as‐formed porous structure possessed high specific surface area, which could provide abundant active adsorption sites. In addition, enormous oxygen vacancies generated on the surface of sensitive materials could efficiently boost the sensing performance at lower working temperature. Therefore, the porous materials synthesized by the self‐templating method can provide sufficient surface oxygen species and contribute to sensing performance, as well as fast response and recovery rate.

In general, the pore structure plays an important role in adjusting the sensing performance. The diffusion efficiency of target molecules inside the ordered pores can be greatly improved compared with the disordered pores. Therefore, the ordered mesoporous structure is much preferred in gas sensing process. In addition, porous materials with interconnected channels facilitate the mass transformation and contribute to the fast response and recovery, which is another important factor to affect their sensing performance. Importantly, the 2D pore structure with rich accessible active sites, high specific surface area and much shorter diffusion pathway are extremely desired for the gas sensing reaction compared with the 1D and 3D pore structure. Particularly, as mentioned above, the tailored ordered mesoporous structure is extremely desired for the gas sensing process due to the unique Knudsen diffusion behavior. The mesoporous structure with tunable pore size is favorable for the gas sensing reaction. Notably, the surface acidic/basic and reactive oxygen species are also highly relevant to the adsorption of target molecules. Thus, the gas sensing performance is strongly dependent on the surface properties of these sensitive materials.

### 2.2 Construction of Heterojunction

For chemiresistive MOS gas sensors, the resistance of sensitive materials can change rapidly when they contact with various gases or different concentrations of the target gases.^[^ ^43^ ^]^ To obtain a superior gas sensing performance of MOS materials, several strategies such as the construction of heterojunction, heteroatom doping, and modification with noble metals have been performed. Among them, construction of heterojunction interface is one of the most important strategies to enhance gas sensing performance, where the Fermi level of the two components is different. To creating intimate electrical contact at the interface, the Fermi levels across the interface can reach the equivalence and the electrons can transfer from the component with high Fermi level to other component with low Fermi level, leading to the charge transfer and further extending the region of charge depletion.^[^ ^44^ ^]^ In addition, other factors including increased interfacial potential barrier energy,^[^ ^45^ ^]^ catalytic activity,^[^ ^46^ ^]^ synergistic surface reactions,^[^ ^47^ ^]^ and increased gas accessibility can also contribute to the enhancement of sensing performance.^[^ ^48^ ^]^ However, there is no complete theory to explain all heterojunction‐gas interactions due to the diversity of structures and reactions. Thus, the fundamental understanding of their specific surface features, energy band structure, and surface catalytic behavior is vital to acquaint the potential sensing mechanism.

In general, the heterojunctions can be classified as p–n junctions, n–n junctions, p–p junctions, and complex heterojunctions (e.g., p–n–p, n–p–n). When two or more different semiconductor materials are in contact with each other, the intimate electrical contacted at the interface can be formed and the Fermi levels of both two components can reach a thermodynamic equilibrium to the same energy, resulting in the formation of interface charge depletion layer, which can further improve the gas sensing performance.^[^ ^49^, ^50^ ^]^ Owing to their small size, porous structure, and high surface areas, the porous semiconductors favor the formation of numerous hetero‐structures and thus are considered as promising sensing materials. As shown in **Table** **1**,^[^ ^51^, ^52^, ^53^, ^54^, ^55^, ^56^, ^57^, ^58^, ^59^, ^60^, ^61^, ^62^, ^63^, ^64^, ^65^, ^66^ ^]^ the sensitive materials with heterojunction structure showed excellent sensing performance toward various gases, including NO_2_, H_2_S, CO, ethanol, acetone, NH_3_, and H_2_, owing to their unique synergistic enhancement effects. However, the microscopic properties of heterojunctions, such as carrier transport, energy bands, structural defects and recombination, and the mechanism of gas–solid chemical reactions between gases and interfaces are still not clear and requires to be further studied.

**Table 1:** Gas sensing performance based on p–n, n–n, and p–p heterojunctions

Materials	Types	Target gas	Concentration	Working temperature	Response	Refs.
In_2_O_3_–NiO	p–n	NO_2_	15 ppm	RT	3	[51]
WO_3_–NiO	p–n	H_2_S	50 ppm	350 °C	170	[28]
WO_3_–CoO–Co_3_O_4_	p–n	CO	200 ppm	400 °C	4.8	[52]
SnO_2_–Cr_2_O_3_	p–n	Trimethylamine	5 ppm	275 °C	209.5	[53]
SnO_2_–NiO	p–n	Ethanol	100 ppm	280 °C	59	[54]
SnO_2_–Co_2_O_3_	p–n	Acetone	5 ppm	450 °C	5.4	[55]
ZnO–ZnCo_2_O_4_	p–n	Trimethylamine	100 ppm	220 °C	5.1	[56]
SmFeO_3_–ZnO	p–n	Acetone	10 ppm	350 °C	45	[57]
NiO–ZnO	p–n	Methane	1000 ppm	380 °C	1.9	[58]
SnO_2_–TiO_2_	n–n	Ethanol	400 ppm	350 °C	102	[59]
WO_3_–TiO_2_	n–n	Acetone	50 ppm	290 °C	14	[60]
WO_3_–ZnO	n–n	NO*_x_*	100 ppm	RT	7.1	[61]
WO_3_–ZnO	n–n	NO_2_	1 ppm	150 °C	167.8	[62]
MoO_3_–SnO_2_	n–n	H_2_S	1 ppm	115 °C	9.2	[63]
Co_3_O_4_/CuO	p–p	Dimethyl methylphosphonate	0.5 ppm	90 °C	8.8	[64]
Fe_2_O_3_/CuO	p–p	NH_3_	200 ppm	RT	4.1	[65]
Zn*_x_* Cu_1−_ *_x_* O*_y_*	p–p	H_2_	100 ppm	300 °C	10.6	[66]

#### 2.2.1 p–n Heterojunctions

It is well known that p–n junction has been regarded as the most efficient structure in catalysis and gas sensor due to the various Fermi‐level of the two semiconductors, and the electrons at the higher energy level can transfer to the lower energy level. Moreover, because of the mismatch of the Fermi level between the two individual components, the transfer of electrons occurred after construction of heterojunctions, and the electrons flow from the higher Fermi level to the lower one. Therefore, the construction of heterojunctions can significantly modify the electronic structure of the materials. The electron holes transfer from the p‐type side to the n‐type side, and the Fermi levels could reach a thermodynamic equilibrium.^[^ ^49^ ^]^ In addition, a space charge layer forms at the interface between n‐type semiconductor (e.g., SnO_2_, ZnO, WO_3_, In_2_O_3_, TiO_2_, MoO_3_) and p‐type semiconductor (e.g., Fe_2_O_3_, NiO, CuO, Co_3_O_4_, Cr_2_O_3_, Mn_2_O_3_). At the same time, the energy band on both sides of the interface is bent, and this can result in a potential barrier, which possess narrowed electronic transmission channel.^[^ ^67^ ^]^ The electron depletion layer width (*X*_n_) in n‐type semiconductor and the electron–hole depletion layer width (*X*_p_) in p‐type semiconductor can be calculated by the following formula, respectively.^[^ ^68^ ^]^

(3) $$X_{n}={\left\{\frac{2\varepsilon_{n}V_{\mathrm{bi}}}{q}\left[\frac{N_{p}}{N_{n}}\right]\left[\frac{1}{N_{n}+N_{p}}\right]\right\}}^{1/2}$$

(4) $$X_{p}={\left\{\frac{2\varepsilon_{p}V_{\mathrm{bi}}}{q}\left[\frac{N_{p}}{N_{n}}\right]\left[\frac{1}{N_{n}+N_{p}}\right]\right\}}^{1/2}$$

where *ɛ*_n_ and *ɛ*_p_ are static dielectric constants, *N*_p_ and *N*_n_ are carrier concentrations in p‐type and n‐type MOS, respectively, and *V*_b_ *_i_* is the potential difference between them. When oxygen is adsorbed on the surface of p–n heterojunction, electrons transport can be inhibited and the effective cross‐sectional area of radial charge conduction in heterojunctions decreased, leading to the increase of their intrinsic resistance.^[^ ^69^ ^]^ After exposing in reducing gas, the target gas reacts with the surface adsorbed oxygen and the electrons can be released to the MOS, leading to the decrease of resistance. Hence, the remarkable resistance variation between initial resistance and final resistance give rise to the enhanced sensing sensitivity.

For example, Dong and Liu^[^ ^51^ ^]^ synthesized In_2_O_3_ decorated‐mesoporous NiO via nanocasting method. Compared with pure mesoporous NiO, the In_2_O_3_‐decorated mesoporous NiO sensors exhibited enhanced sensing performance towards NO_2_ with excellent selectivity in the presence of interfering gases (i.e., CO, NH_3_, ethanol, methanol, formaldehyde, toluene and acetone). It shows that the formation of In_2_O_3_–NiO p–n junctions can modify the width of space‐charge layer, and the hole depletion layer can be formed owing to the higher Fermi level of In_2_O_3_ than NiO. Furthermore, the recombination of electrons in the n‐type In_2_O_3_ and hole in p‐type NiO occurred in the accumulation layer, resulting in the decrease of carrier and the increase of the resistance. When the semiconductor was exposed to NO_2_, NO_2_ molecules could trap the electrons to generate more electron holes, leading to the decrease of resistance.

**Figure 4:**
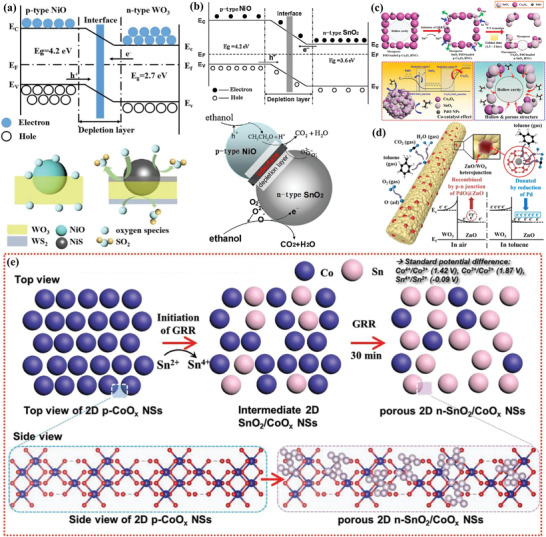
a) p–n heterojunction structural mesoporous NiO/WO_3_ composites for H_2_S sensing mechanism. Adapted with permission.^[^ ^70^ ^]^ Copyright 2019, American Chemical Society. b) Energy band structure of p–n heterojunction NiO/SnO_2_ and their sensing mechanism toward reducing gas. Adapted with permission.^[^ ^54^ ^]^ Copyright 2012, Elsevier. c) p–n heterojunction formation principle and sensing operating mechanism. Adapted with permission.^[^ ^55^ ^]^ Copyright 2017, American Chemical Society. d) Acetone sensing mechanism for heterojunction structural Pd@ZnO‐WO_3_ nanofibers. Adapted with permission.^[^ ^41^ ^]^ Copyright 2016, American Chemical Society. *E*_c_: lower level of conduction band; *E*_F_: Fermi level; *E*_v_: upper level of valence band. e) The formation route of heterogeneous porous SnO_2_/CoO*_x_* nanosheets via galvanic replacement reaction. Adapted with permission.^[^ ^71^ ^]^ Copyright 2019, Wiley.

In addition, the surface reaction between p–n heterojunction and target gas is also contributed to the excellent sensing performance. Typically, Xiao et al.^[^ ^70^ ^]^ rationally designed the mesoporous p–n heterojunction semiconductors based on n‐type WO_3_ and p‐type NiO via evaporation‐induced multicomponent coassembly strategy. The sensing mechanism relies on the electron‐depleted surface region due to the adsorption of oxygen species before exposure in H_2_S gas, and the absorbed oxygen species are reduced and the trapped electrons can be released to the conduction band, resulting in the decrease of resistance once contacting H_2_S gas (**Figure** **4**a). It was found that both SO_2_ and sulfides (WS_2_ and NiS) were formed during the gas sensing process. Such a process helps to reduce the resistance of the nanocomposites, leading to the enhancement of the gas sensing performance. In addition, the mesoporous structure with high porosity and numerous active sites can facilitate the gas diffusion and adsorption. The templating synthesis based on PEO‐*b*‐PS with different molecular weights gave rise to mesoporous materials with tunable pore structure and large pore size up to ≈30 nm as well as ultralarge window size (12–16 nm). It was found that the ultrasmall NiO nanocrystals were embedded into the crystalline frameworks of mesoporous WO_3_. As a result, the binary metal oxide mesoporous semiconductors with rich p‐n heterojunctions exhibited excellent sensing performance towards H_2_S.

Particularly, Nguyen et al.^[^ ^52^ ^]^ reported mesoporous cobalt tungsten oxide heterostructured nanotoroids for the detection of three reduction gases (CO, H_2_, NH_3_). Importantly, the content of p–n junction determined the main carriers in the semiconductors, and the ratio of Co to W was 2:1, indicating that the heterostructures might mainly exhibit the properties of p‐typed semiconductor. Actually, the resistance of the sensors increased after the injection of reduction gas, demonstrating this kind of materials showed the p‐typed semiconductor properties. It was demonstrated that when the sensor was exposed in air, the adsorbed oxygen could trap the electrons from the semiconductor and the additional holes in the valence bond could be generated, leading to the decrease of resistance. When the reduction gas was introduced, the adsorbed oxygen could react with the reduction gas and electrons returned to the conduction band of semiconductor and decreased the density of hole carriers, resulting in the increase of the resistance. Typically, in the Co‐W oxide p–n junctions, n‐typed WO_3_ facilitated the chemisorption of oxygen, and the p‐typed CoO acted as catalytic active sites. Therefore, the increased concentration of adsorbed oxygen nearby the CoO components can provide more active sites to accelerate the gas‐sensing oxidation reactions, contributing to the enhancement of the gas‐sensing performance. Park et al.^[^ ^53^ ^]^ also indicated that the minority of p‐typed semiconductor in the p–n junctions could act as the catalytic active sites to the target gas. It showed that the decoration of discrete configuration of p‐typed Cr_2_O_3_ nanoparticles in n‐type SnO_2_ could remarkably improve their sensitivity and selectively towards trimethylamine due to the formation of p–n junction and the surface catalytic promotion effect of Cr_2_O_3_. The surface defects can be also formed when two different components contacted with each other, and the defects sites are conducive to the adsorption of specific target gases. Therefore, more gas molecules can participate in the surface adsorption‐catalytic reaction, resulting in the narrower hole accumulation layer and larger resistance. For example, the crystal lattice parameters of NiO and SnO_2_ were different, and abundant surface defects could be created at the grain boundary of p–n junction (Figure 4b),^[^ ^54^ ^]^ which could form an abundant heterogeneous interface to provide active sites for ethanol sensing.

Interestingly, the type of semiconductors sensitive materials can be adjusted by rationally modifying the synthesis strategy. In this regard, Jang et al.^[^ ^55^ ^]^ reported the porous metal oxide polyhedron and changed from p‐type to n‐type via galvanic replacement reaction (GRR) (Figure 4c). In this work, Pd nanoparticles (NPs) loaded p‐type Co_3_O_4_ porous cubes (p‐Co_3_O_4_) were obtained by using MOFs as the sacrificial scaffold, and it could further transfer and load into n‐type SnO_2_ (n‐SnO_2_) with porous polyhedron structures via p–n phase transition. Similarly, Kim and co‐workers^[^ ^41^ ^]^ designed Pd‐embedded ZnO nanocubes via MOFs template strategy, and it could be regarded as active species to load into the WO_3_ nanofibers through electrospinning, and the obtained nanofibers (Pd@ZnO‐WO_3_) exhibited superior sensing performance toward toluene vapor (Figure 4d). By controlling the conditions of GRR, a series of semiconductor materials with various conductive properties can be obtained (Figure 4e).^[^ ^71^ ^]^ It indicated that the nanocomposite consisted of p‐Co_3_O_4_ (major component) and n‐SnO_2_ (minor component) possessed poor sensing properties. Therefore, the p–n junctions with n‐type sensing matrix as the majority group and p‐type catalytic components as the minority group is favorable in design of high‐performance sensing materials. Furthermore, porous 2D heterogeneous oxide (e.g., SnO_2_/CoO*_x_*) can be also fabricated through a 2D oxide exfoliation approach combined with a fast GRR approach.^[^ ^71^ ^]^ The controlled GRR process promised the hybrid materials with small SnO_2_ grain size, high porosity, and numerous p–n heterojunctions, providing numerous oxygen adsorption sites for excellent formaldehyde sensing.

In addition, other p–n junctions such as ZnO–ZnCo_2_O_4_,^[^ ^56^ ^]^ SmFeO_3_–ZnO,^[^ ^57^ ^]^ NiO–ZnO^[^ ^58^ ^]^ and NiO–SnO_2_^[^ ^72^ ^]^ were also reported, exhibiting significantly improved gas sensing performance. The construction of p–n junctions not only facilitates the formation of electron depletion layer but also increases the barrier height. Moreover, the oxygen vacancies originated from doping hetero‐ metal atoms in the lattice of metal oxides can act as the gas molecule adsorption sites and catalytic active sites, which can dramatically enhance their sensing performance.

#### 2.2.2 n–n or p–p Heterojunctions

**Figure 5:**
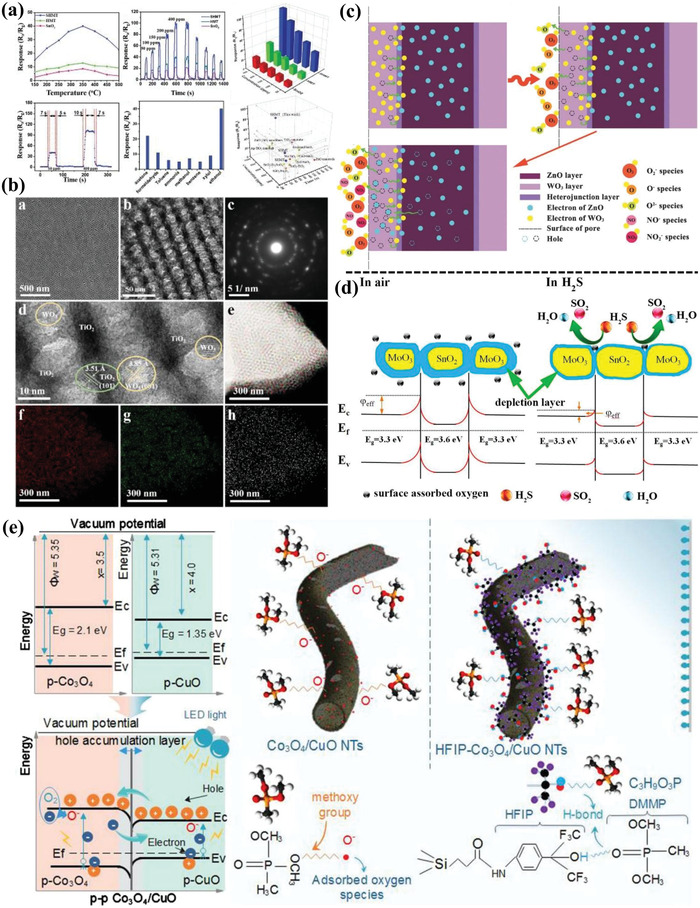
a) Efficient ethanol sensing behavior of hierarchical branched mesoporous TiO_2_–SnO_2_ semiconducting heterojunctions. Adapted under the terms of the Creative Commons CC‐BY license.^[^ ^59^ ^]^ Copyright 2019, The Authors. Published by Wiley. b) Morphology and structure characterization of the highly crystallized ordered mesoporous TiO_2_/WO_3_ heterojunctions. Adapted with permission.^[^ ^60^ ^]^ Copyright 2020, Elsevier. c) NO*_x_* gas sensing mechanism on ordered mesoporous WO_3_/ZnO n–n heterojunction. Adapted with permission.^[^ ^61^ ^]^ Copyright 2018, Wiley. d) Energy band structure and H_2_S sensing mechanism of the porous MoO_3_/SnO_2_ n–n junctions nanofibers. Adapted with permission.^[^ ^63^ ^]^ Copyright 2019, American Chemical Society. e) Energy band structure variation and dimethyl methylphosphonate sensing mechanism of the porous Co_3_O_4_/CuO p–p heterojunctions nanotubes. Adapted with permission.^[^ ^64a^ ^]^ Copyright 2020, Elsevier.

For n–n or p–p heterojunctions, electrons can be transferred from the high Fermi level side to the low Fermi level side, forming an electron accumulation layer on one side and an electron depletion layer on the other side. Meanwhile, this depletion layer can be further depleted by adsorbed oxygen on the semiconductor surface, resulting in the narrower conductive channel and increased resistance, which is favorable for the enhancement of sensing response. Typically, Zhao et al.^[^ ^59^ ^]^ synthesized the hierarchical branched mesoporous TiO_2_–SnO_2_ nanocomposites with well‐defined n–n heterojunctions through evaporation induced oriented coassembly strategy for highly efficient ethanol sensing (**Figure** **5**a). Due to the abundant surface hydroxyl groups of SnO_2_ NCs, the SnO_2_ NCs can easily interact with PEO segments of the PEO‐*b*‐PS template via hydrogen bonds. Therefore, SnO_2_ NCs were homogeneously distributed in the mesoporous TiO_2_ matrix, forming numerous n–n heterojunctions. Typically, the n–n heterojunctions could be formed when the two semiconductors are contacted via the band alignment. The electrons can transfer from TiO_2_ to SnO_2_, forming the accumulation layer on SnO_2_ rather than a depletion layer. Hence, the accumulation layer can be depleted by subsequent oxygen adsorption, which increase the potential energy barrier at the interface and facilitate the oxygen adsorption on SnO_2_ surface, resulting in excellent sensing performance. In addition, the unique hierarchical branched mesoporous structure with a large pore size, abundant active sites and much shorter diffusion distance are particularly favorable for the diffusion and rapid transfer of gas molecules across its surface, resulting in the higher sensitivity and shorter response time. Similarly, Wang et al.^[^ ^60^ ^]^ also synthesized the highly crystallized mesoporous TiO_2_/WO_3_ n–n heterojunctions through a novel “acid‐base pair” adjusted solvent evaporation induced self‐assembly strategy. The obtained materials possessed large pore size (≈21.1 nm), highly crystalline frameworks and large specific surface area (≈ 98 m^2^ g^−1^) and exhibited superior gas sensing performance towards acetone at 290 °C (Figure 5b). Typically, the high surface area can provide abundant active sites to adsorb oxygen species on the sensing layer of mesoporous walls, and the interconnected mesopores facilitate the diffusion of acetone molecules. Importantly, the unique TiO_2_/WO_3_ n–n heterojunctions contributed to the formation of electron depletion layer, leading to improved sensing response and excellent response/recovery performance.

Additionally, the gas sensing properties are also highly related with the microstructure of the n–n heterojunctions. Han et al.^[^ ^61^ ^]^ designed ordered mesoporous WO_3_/ZnO materials by electroless plating. In this composite, layered WO_3_ film was assembled on the surface of mesoporous ZnO, resulting in a unique core–shell heterojunction structure. Due to the higher Fermi level of WO_3_ than that of ZnO, the electrons in WO_3_ can flow into ZnO layer to achieve an equilibration of the Fermi level (Figure 5c). When the sensors were exposed in air, the adsorbed oxygen on the WO_3_ layers could capture electrons from the conduction band to form oxygen species, resulting in distinct electrons concentration difference around the n–n heterojunction interface. During sensing performance evaluation, the target NO*_x_* molecules could capture the electrons in the conduction band of WO_3_ layer, and the electrons concentration difference between two sides of the n–n heterojunctions was further enlarged, causing the migration of electrons through the n‐n hetrojunctions from ZnO to WO_3_. Therefore, the resistance sharply decreased when the sensors were exposed in NO*_x_* gas. Nevertheless, when the sensors were exposed in reduction gases (such as NH_3_), NH_3_ molecules may react with the chemisorbed oxygen, and the electrons are released back to the conduction band of WO_3_, resulting in the increase of electron concentration and the decrease of the sensor resistance.

By contrast, Sun et al.^[^ ^62^ ^]^ synthesized the ordered mesoporous WO_3_/ZnO nanocomposites with ZnO nanoparticles decorating the framework of WO_3_. Therefore, the chemisorbed oxygen species can form a depletion layer on the interface of both oxides. At the interface of the two metal oxides, electrons transferred from WO_3_ to ZnO to form an additional depletion layer of charge carriers, and the resistance of the composites decreased when exposing in air. It was found that the sensing properties were strongly dependent on the structures and components of the sensitive materials. In addition, the porous MoO_3_/SnO_2_ nanoflakes with n–n junctions showed superior sensing performance toward H_2_S gas (Figure 5d).^[^ ^63^ ^]^ Due to the larger work function of MoO_3_ (5.3 eV) than that of SnO_2_ (4.7 eV), the electrons tend to transfer from SnO_2_ to MoO_3_ in the nanoscale n–n junctions. When the obtained materials are exposed in air, the adsorbed oxygen can capture the electrons from the conduction band, leading to the increase of effective energy barrier. In H_2_S atmosphere, the captured electrons can transport to the conduction band of MoO_3_ and SnO_2_ owing to the reaction between oxygen species and H_2_S molecules, resulting in the decrease of the potential energy barrier, which is beneficial to achieving a superior H_2_S sensing performance. In order to verify the contribution of n–n junctions, physically mixed materials with the same ratio of Mo to Sn oxides were prepared. However, the physical mixture exhibited inferior H_2_S sensing performance compared with the porous MoO_3_/SnO_2_ nanoflakes. It implied that the tightly contacted interfaces between MoO_3_ and SnO_2_ in nanoscale contributed the excellent gas sensing performance.

Additionally, the construction of p–p heterojunctions is also in favor of enhancing gas sensing performance. Lupan et al.^[^ ^66^ ^]^ reported non‐planar nanoscale Zn*_x_* Cu_1‐_ *_x_* O*_y_* nanocrystals with mixed phases, and the typical p–p junctions (i.e., Cu_2_O:Zn and CuO:Zn) were obtained by rapid thermal annealing method. Due to the mismatched work function of Cu_2_O:Zn and CuO:Zn, the electron holes can transfer from Cu_2_O:Zn to CuO:Zn, forming a hole accumulation layer (HAL) at their interface. In air, the absorbed oxygen molecules can capture electrons from the surface of CuO:Zn, resulting in the decrease of resistance. Upon exposure to H_2_, the released electrons recombined with electron holes from the HAL region and the resistance was increased significantly. In addition, the p–p junction formed by heteroatom doping technique can also enhance their gas sensing performance. For example, Bhuvaneshwari et al.^[^ ^65^ ^]^ reported porous Fe‐doped CuO materials for superior NH_3_ sensing performance. The excellent performance was attributed to the incorporation of Fe^2+^ in the CuO lattice and the consequent variation in its charge carrier (i.e., holes) concentration, as well as the electronic interaction between the Fe and CuO. Similarly, the visible light irradiation can enhance the gas sensing performance of Co_3_O_4_/CuO heterojunctions towards dimethyl methylphosphonate (DMMP), a sarin nerve agent simulant (Figure 5e).^[^ ^64a^ ^]^ First, the formation of p–p heterojunctions can reduce the potential energy barriers, and the resistance of the sensing materials is decreased when exposing in air due to the formation of chemisorbed oxygen species. Additionally, light irradiation can further reduce the resistance of the sensors due to the excitation of electrons from the valence band to the conduction band, leaving a hole in the valence band. Therefore, the electrons can transfer to the sensing material and reduce the conductivity of the sensor in reducing DMMP atmosphere.

Generally, the sensitive response of MOS is based on the resistance change of the materials when exposed in air and target gas molecules. Therefore, a variety of methods can be employed to improve the sensing performance of MOS materials, such as tuning the composition, optimizing the microstructures, modifying the component ratio, constructing junctions and creating surface defects, which can adjust their electronic structure, interfacial potential barrier energy, synergistic surface reactions, and etc.

Unlike the normal p–n and n–n, or p–p structure, the n–p–n or p–n–p structures are also important in electronic devices. Notably, the band structure, surface properties, synergetic depletion effect, and interfacial charge transfer are more complicated. Typically, An et al. fabricated the three‐layered ZnO–CuO–SnO_2_ hybrid n–p–n heterojunctions by three‐step chemical vapor deposition on SiO/Si substrates and further investigated the *I–V* characteristics.^[^ ^64b^ ^]^ Notably, CuO hollow microspheres with branching lines, ZnO nanowires, and SnO_2_ nanorods establish the n–p–n heterojunctions, and the *I–V* characteristics exhibit the rectifying behavior, promising their excellent sensing performance. In addition, the theoretical macroscopic method is also performed to investigate the n–p–n typed SnO_2_–CuO–SnO_2_ heterojunctions in H_2_S sensing process.^[^ ^64c^ ^]^ The 3D “SnO_2_ wire/CuO shell” model was established, and while these two configurations exhibited nearly similar electrical resistances in H_2_S atmosphere in comparison with “mono‐wire” and thin bilayer models. Moreover, Singh et al. fabricated n‐SnSe_2_/p‐SnO/n‐SnSe heterojunctions by thermal evaporation approach, and gas sensors based the n–p–n typed heterojunctions showed excellent sensing performance to NO_2_ gas at room temperature with a high sensing response (256% for 5 ppm NO_2_) and the low detection limit of 115 ppb, and the sensors also showed fast response of 34 s.^[^ ^64d^ ^]^ It was found that the n‐typed SnSe could reduce the resistance due to its much better conductivity, which could further boost the sensing response of NO_2_ and contribute to the much lower detection limit. It can be mainly attributed to the synergistic effect of physisorption and charge transfer. However, the overall reports about p‐n‐p typed heterojunctions are relatively limited, and the detailed sensing mechanisms are still unclear, therefore, more work should be done to investigate these novel heterojunctions.

### 2.3 Modification with Noble Metal

Recently, the noble metal‐based sensing materials have received tremendous research interests due to their fascinating intrinsic properties, including high reactivity, unique catalytic activity and small size feature. Although considerable efforts have been devoted to disclosing detailed mechanisms, it is also highly desired to explore a general strategy for bottom‐up design of gas‐specific sensing materials. Thus, precise and controllable incorporation of noble metal particles in mesopore channels requires an elegant chemical synthesis and a nanoengineering strategy.

The modification of MOS with noble metals can significantly improve their gas‐sensitive performance, which can be explained from two aspects of “electronic sensitization” and “chemical sensitization.” For the electronic sensitization, the Schottky barrier can form at the interface of semiconductor materials and noble metal when they are in contact. Since the noble metals possess strong oxygen adsorption characteristics, and the adsorbed oxygen species on the noble metal surface can capture electrons from the semiconductor, leading to the much thicker electron depletion layer near the surface of the semiconductor. Therefore, the conductivity of the sensitive materials decreases and the resistance increases. When reducing gas is injected, the reaction between metal oxides and target gas can return electrons to the sensitive materials, resulting in the decrease of material's resistance. In addition, the surface potential energy is nonuniform and the change of adsorbed potential energy can be also uneven. Therefore, when the thermal motion energy exceeds the boundary barrier energy, the absorbed species can transfer from their original positions to the surface of the catalyst, which is the phenomenon of “chemical sensitization.” In gas sensing reaction, the target gas or oxygen molecules can be adsorbed, then accumulated and spilled over to the surface of semiconductors. The noble metals nanoparticles in the surface of semiconductor materials can act as the active sites to promote their gas sensing performance. Moreover, the metal nanoparticles also exhibit excellent catalytic properties, which is highly relevant with the nanoparticle size.

**Figure 6:**
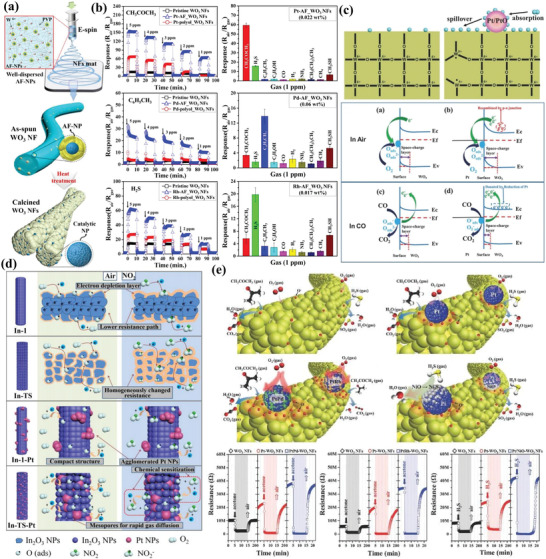
a) Electrospinning synthesis route for mesoporous WO_3_ nanofibers functionalized by bioinspired catalytic NPs (i.e., Pt, Pd, and Rh). b) Dynamic gas‐sensing behaviors of noble‐metal loaded WO_3_ nanofibers toward various gases. Adapted with permission.^[^ ^73^ ^]^ Copyright 2016, American Chemical Society. c) The adsorbed oxygen species evaluation in the Pt‐sensitized mesoporous WO_3_ and potential CO sensing mechanism. Adapted with permission.^[^ ^74^ ^]^ Copyright 2018, Wiley. d) NO_2_ sensing mechanism on Pt‐loaded In_2_O_3_ mesoporous nanofibers. Adapted with permission.^[^ ^76^ ^]^ Copyright 2018, Elsevier. e) The sensing mechanism of various bimetallic Pt‐based NPs (PtM, where M = Pd, Rh, and Ni) encapsulated mesoporous WO_3_ nanofibers toward acetone or hydrogen sulfide and dynamic resistance change transients. Adapted with permission.^[^ ^78^ ^]^ Copyright 2017, Wiley.

Inspired by the noble metal doping strategy, Kim et al.^[^ ^73^ ^]^ synthesized metal nanoparticles decorated mesoporous WO_3_ nanofibers for the detection of trace biomarkers in exhaled breath. Particularly, apoferritin‐encapsulated catalytic NPs (AF‐NPs) were firstly prepared, and then the apoferritin‐encapsulated Pt, Pd, and Rh NPs (Pt‐AF, Pd‐AF, and Rh‐AF) were synthesized as catalysts for the sensitization of WO_3_ nanofibers, respectively (**Figure** **6**a). The Pt‐AF_WO_3_ materials showed superior sensing performance towards acetone, and the adsorbed oxygen on metallic Pt NPs could spill over to the WO_3_ matrix, leading to the increase of chemisorbed oxygen species on the WO_3_ nanofibers. Therefore, the Pt‐AF_WO_3_ sensors exhibited higher baseline resistance. In the presence of acetone vapor, oxidation of acetone by the adsorbed oxygen in the surface sensitive layer caused the dramatic decrease of resistance of sensors and improved acetone sensitivity (Figure 6b). While Pd and Rh can be also regarded as electronic sensitizers to change their oxidation state by adsorption of oxygen in air. The p‐typed PdO and Rh_2_O_3_ can be formed at high temperatures during sensing process, and the formation of p–n junctions can induce a higher baseline resistance. In reducing gas atmosphere (e.g., H_2_S or toluene vapor), the electrons were donated back to the conduction band, resulting in remarkable resistance changes, contributing to the improvement of gas sensing performance. Thus, in the MOS‐based gas sensing system, nanosized noble metals can play dual function roles in enhancing the sensing performance.

Besides, the noble metals can be also regarded as the catalytic sites to react with target gases. In this regard, Ma et al.^[^ ^74^ ^]^ synthesized Pt NPs sensitized mesoporous WO_3_ via a straightforward coassembly strategy where ultrafine Pt NPs were in situ homogeneously generated in mesopores due to the confinement effect. The obtained materials exhibited uniform pore size (≈ 13 nm), high specific surface area (128 m^2^ g^−1^), large pore volume (0.32 cm^3^ g^−1^) and ultrasmall Pt NPs (≈4 nm). As a result, the Pt‐decorated mesoporous WO_3_ exhibited excellent catalytic sensing response to CO gas. The Pt NPs not only facilitated the oxidation of CO, but also improved the adsorption‐desorption reactions of oxygen. It was found that the content of adsorbed oxygen (O^−^ and O_2_^−^) for mesoporous WO_3_/Pt‐0.5 was much higher than that in pure mesoporous WO_3_, indicating the spill‐over effect of noble metal additives on semiconductor WO_3_ (Figure 6c). In addition, the Schottky junction can be formed and the oxidized Pt species can be regarded as the p‐type semiconductor, which facilitates the formation of p–n junctions at the interface between PtO*_x_* and WO_3_.

In addition, the d orbitals of noble metals are not filled, which can facilitate the adsorption and dissociation of target gas molecules. For example, Lu et al.^[^ ^75^ ^]^ reported Pt‐loaded mesoporous WO_3_ materials for the detection of NH_3_ gas, and Pt NPs could decrease the activation barrier and promote the dissociation of N—H bond. In addition, the adsorbed NH_4_^+^ ions could spill‐over on the surface, facilitating the reaction between NH_3_ molecules and surface oxygen species. The mesoporous structure also favored for the diffusion and transport NH_3_ into the sensing layer, further enhanced the gas sensitivity of the materials.

It is well known that the incorporation of noble metal in metal oxides semiconductors can significantly decrease their operating temperature, which is highly desired for the design of advanced gas sensing devices with low energy consumption. Typically, the gas sensing performance evaluation of mesoporous Pt/In_2_O_3_ nanofibers also indicated that the introduction of Pt NPs significantly decreased the operating temperature to room temperature due to the high dispersion state and chemical sensitization of Pt NPs.^[^ ^76^ ^]^ The formation of Pt–In_2_O_3_ heterostructure provided abundant active sites for the dissociation of oxygen species into surface oxygen species and adsorption of NO_2_ molecules, resulting in the enhanced sensing performance (Figure 6d). Similarly, Ag‐doped ordered mesoporous TiO_2_/SnO_2_ nanocomposites also confirmed the important role of noble metal in improving the gas sensing performances.^[^ ^77^ ^]^ The chemical sensitization and catalytic oxidation (spill‐over effect) of Ag NPs also favored the enhancement of gas sensing response. Moreover, different from Pt NPs, the work function of Ag NPs was lower than TiO_2_ and SnO_2_, therefore, the electrons in Ag NPs would flow to the TiO_2_–SnO_2_ interface, resulting in a negatively charged accumulated layer around the contact interface, and this facilitated the dissociation of adsorbed oxygen. Simultaneously, Ag NPs tended to form Ag_2_O in air, and when the composite materials were exposed in reductive ethanol gas, the redox reaction between Ag_2_O and Ag would release the trapped electrons back to TiO_2_ and SnO_2_, leading to superior sensing response.

In addition to single noble metal NPs, bimetallic NPs have been also employed to improve and tune the sensing performance of semiconductor metal oxides. In this regard, Kim et al.^[^ ^78^ ^]^ reported a facile and rational method to synthesize a new class of active Pt‐based bimetallic (PtM, where M = Pd, Rh, and Ni) NPs. Transmission electron microscopy (TEM) observation showed well‐dispersed PtM NPs with small size (<3 nm), which were supported on the mesoporous WO_3_ nanofibers. Although the loading amount of PtM NPs was pretty low, they exhibited excellent sensing performance towards acetone and hydrogen sulfide in the exhaled breath (Figure 6e). It is worth noting that the Pt‐based alloys can effectively dissociate the molecular oxygen and analyte, accelerating the chemical reaction compared to Pt NPs. To gain more insight, the baseline resistance of pristine WO_3_ NFs, Pt‐WO_3_ NFs, PtPd‐, and PtRh‐WO_3_ NFs were investigated, and the resistance of PtPd‐WO_3_ NFs (38.3 MΩ) was much higher than the WO_3_ NFs (10.4 MΩ) and Pt‐WO_3_ NFs (22.9 MΩ). In addition, it was found that p‐type PtO*_x_*, Rh_2_O_3_, and PdO could be generated on the WO_3_ NFs during calcination at high temperatures, and the formation of p–n junctions contributed to the increase of baseline resistance. When the sensor was exposed to the reductive gases, the depletion layer was significantly decreased by returning electrons to WO_3_, leading to a dramatic change in resistance. In addition, the synergistic effect between bimetallic NPs and porous matrix can also improve the sensing selectivity due to their modified surface electronic structure and catalytic effect.

As mentioned above, the enhancement effect of sensing performance by noble metals can be summarized as follows: 1) oxygen and target molecules can be readily adsorbed in sensing materials, reducing the adsorption activation energy; 2) noble metals act as the catalytic sites to effectively dissociate the molecular oxygen and analyte; 3) the Schottky barrier can form at the metal–metal oxides contact interface, leading to the thicker depletion regions; 4) the spillover effect reduces the activation energy of the reaction; 5) the formed oxidation state of noble metals facilitates the formation of p‐n junctions with oxides matrix, improving their sensing performance.^[^ ^79^ ^]^

### 2.4 Nonmetal Heteroatom Doping Engineering

Element doping is another effective way to modulate the electronic structure of semiconductors and thus influences their versatile functionalities and physicochemical properties (e.g., conductivity, bandgap, and internal defect), which can further affect the gas sensing performance. Typically, the doping of heteroatoms such as C, N, Si, and S into the framework of semiconductor metal oxides can significantly modify their properties (e.g., intrinsic ionic conductivity) and energy band structure, which enhance the surface adsorbed oxygen and interfacial reaction efficiency to accelerate surface adsorption‐catalytic reaction (**Table** **2**).^[^ ^80^, ^81^, ^82^, ^83^, ^84^, ^85^, ^86^, ^87^, ^88^, ^89^, ^90^, ^91^ ^]^ Compared with traditional metal doping, non‐metal elements can provide amorphous species to enhance the thermal stability, and it can also improve gas sensing selectivity, sensitivity and reduce operation temperature. Therefore, it is vital to elaborately design semiconductor materials via nonmetal heteroatom doping engineering.

**Table 2:** Gas sensing performances of gas sensors based on heteroatom doping

Materials	Type	Target gas	Concentration	Working temperature	Response	Refs.
C‐doped TiO_2_	Carbon doped	Ethanol	5 ppm	150 °C	1.7	[80]
C‐doped TiO_2_	Carbon doped	n‐pentanol	100 ppm	170 °C	11.2	[81]
C‐doped WO_3_	Carbon doped	Acetone	5 ppm	300 °C	8	[82]
C‐Fe‐doped WO_3_	Carbon doped	Acetone	10 ppm	300 °C	18	[84]
C‐doped WO_3_	Carbon doped	Acetone	10 ppm	350 °C	13.5	[85]
C‐doped ZnO	Carbon doped	Ethanol	200 ppm	RT	2	[86]
N‐doped TiO_2_	Nitrogen doped	Acetone	250 ppm	400 °C	17.6	[19]
N‐doped WS_2_/C	Nitrogen doped	NO_2_	5 ppm	RT	48.2%	[95]
N‐doped SnO_2_	Nitrogen doped	NO_2_	5 ppm	80°C	155	[100]
N‐doped ZnO/C	Nitrogen doped	NO_2_	20 ppm	200°C	265.8	[101]
N‐doped CoS_2_	Nitrogen doped	NO_2_	100 ppm	RT	62.3	[102]
Si‐doped WO_3_	Silica doped	Acetone	600 ppb	400 °C	4.5	[87]
Si‐doped WO_3_	Silica doped	Acetone	50 ppm	300 °C	216	[88]
Si‐doped MoO_3_	Silica doped	NH_3_	1 ppm	400 °C	2.5	[89]
Si‐doped ZnO	Silica doped	Acetone	50 ppm	240 °C	32	[39]
S‐doped SnO_2_	Sulfur doped	NO_2_	100 ppm	RT	57.38	[90]
S‐doped SnO_2_	Sulfur doped	NO_2_	5 ppm	50 °C	600	[91]

#### 2.4.1 Carbon‐Doped Porous MOS

**Figure 7:**
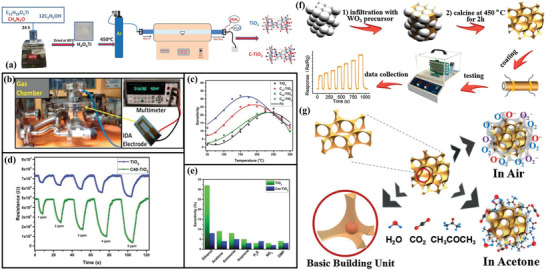
a) Synthesis process of C‐doped TiO_2_. b) The gas sensor test system and device. c) Gas sensing behavior under various temperatures toward 1 ppm ethanol. d) Ethanol gas sensing performance under various concentrations at 150 °C. e) Gas sensing selectivity (1 ppm) at 150 °C. Adapted with permission.^[^ ^80^ ^]^ Copyright 2019, Wiley. f) The synthesis process of 3D ordered macroporous/mesoporous C‐doped WO_3_ and acetone sensing testing route. g) Acetone sensing mechanism on 3D C‐doped WO_3_ sensor. Adapted with permission.^[^ ^85^ ^]^ Copyright 2019, Elsevier.

Carbon as an important nonmetallic element plays a crucial role in catalysis and energy conversion, and it can be also regarded as impurity component in controlling the micro/nanostructure and properties of sensing materials, including local electron location, surface active sites and conductivity. For instance, Raghu et al.^[^ ^80^ ^]^ synthesized C‐doped TiO_2_ with rich (101) facets via a facile mild thermal decomposition technique based on urea as carbon source (**Figure** **7**a), and it showed superior ethanol sensing performance (34.8%) than pure TiO_2_ (8.5%) at low power consumption. Benefitting from the substitutional and interstitial C‐doping into the lattice of anatase TiO_2_, abundant active sites, oxygen vacancies and chemisorbed oxygen species can be formed to accelerate interfacial catalytic reaction (Figure 7b–e). Interestingly, graphitic carbon was found to be capable of enhancing the electrical conductivity and reducing the bandgap, which can decrease the operating temperature from 300 °C to 150 °C in ethanol sensing. Similarly, Zhao et al.^[^ ^81^ ^]^ reported a facile template‐free solvothermal method to synthesize C‐doped TiO_2_ nanoparticles (ca. 30 nm) without additional carbon source, and the obtained materials showed excellent alcohol sensing performance and enhanced sensitivity with the increase of carbon chain lengths. By contrast, C‐doped TiO_2_ exhibited superior n‐pentanol sensing response (11.12 vs 100 ppm), about 5.4 times higher than that in pure TiO_2_. It demonstrates that the trace‐carbon doped can not only enhance the electrical conductivity and accelerate the electron transfer, but also increase the surface adsorbed oxygen to reduce the operating temperature and increase the sensing response.

Additionally, WO_3_ as an important sensitive material has been widely used in efficient gas sensing toward various gases, such as ethanol, acetone, H_2_S and so on. Similarly, carbon elements can also regulate the sensing performance of porous WO_3_ via heteroatomic doping engineering. Typically, Xiao et al.^[^ ^82^ ^]^ reported facial cotton fiber templating method to synthesize C‐doped WO_3_ materials, and the X‐ray photoelectron spectroscopy (XPS) results showed that the binding energy of W4f_7/2_ peak of C‐doped WO_3_ shifted to low binding energy compared with the undoped WO_3_. It indicated that the carbon doped into the lattice of WO_3_ could lead to the increase of electronic density near its adjacent W atoms. In addition, the W4f_7/2_ peaks in C‐doped WO_3_ became wider, indicating that increased amount of oxygen vacancies in WO_3_ lattice was formed, and this was particularly important to improve response and selectivity toward acetone.

It is worth noting that C‐doped WO_3_ arrays showed enhanced sensing performance toward room temperature detection of hydrogen.^[^ ^83^ ^]^ It was demonstrated that the doped carbon could exist as carbonate species, which would be another beneficial factor for the improvement of sensing response to hydrogen. Specifically, in the doped metal oxides, C atoms were mainly bonded to O atoms to form CO‐like species, which accounted for the total energy reduction and subsequently induced an effective bandgap reduction. Therefore, the activation energy for gas sensing could be significantly reduced, which contributed to the improvement of hydrogen sensing response.

In fact, the incorporation of carbon into WO_3_ nanomaterials showed a specific crystal phase which was beneficial for their gas sensing performance.^[^ ^84^ ^]^ Typically, Wang et al.^[^ ^85^ ^]^ synthesized the C‐doped WO_3_ materials by coupling carbon sphere templates with post‐heat treatment method, and the obtained sensing materials were found to be *ε*‐phase WO_3_, which exhibited high sensing selectivity to acetone (Figure 7f). The carbon atoms derived from the carbon spheres might diffuse into the interstitial position of WO_3_ lattice, forming the unique *ε*‐phase WO_3_. Due to the above unique properties, the obtained C‐doped WO_3_ materials exhibited high response to acetone at the concentration of 0.2–10 ppm with high selectivity toward methanol, ethanol, toluene, CO, NO, and NH_3_. It demonstrated that the porous structure with small *ε*‐WO_3_ particles size (12–22 nm) less than double thickness of the space‐charge layer (60 nm for WO_3_ sat 300 °C), and the hollow structure with proper pore size (6.2 nm) were contributed to the excellent gas sensing performance (Figure 7g). In addition, they also synthesized the three‐dimensionally ordered macro/mesoporous C‐doped WO_3_ materials with different pore sizes ranged from 205 to 730 nm by tuning polystyrene (PS) microspheres template. They have investigated the relationship between the pore sizes and the gas sensing properties comprehensively according to the depletion layer theory. When the size of basic structural units was less than the double thickness of the space‐charge layer (Debye length 2*δ*), the whole semiconductor would be depleted, and the changes in oxygen species concentration would influence the whole materials. If the sphere diameter is bigger than 2*δ*, the sensing mechanism will be controlled by the grain boundaries.

Moreover, carbon‐doped ZnO microspheres were prepared through a facile hydrothermal process and exhibited superior UV‐activated room‐temperature gas sensing activity for the detection of ethanol.^[^ ^86^ ^]^ It showed that the presence of sp^2^ carbon could improve the separation extent and restrain the recombination of the photo‐induced electrons and hole carriers in C‐doped ZnO, which was beneficial to improve the photoelectrical activity of samples. And upon exposure the materials under UV light, the photoinduced oxygen ions were created which could react with adsorbed oxygen, then the highly reactive species were responsible for the excellent room‐temperature gas sensitivity. In conclusion, the incorporation of carbon into the porous metal oxides semiconductors can modify their bandgap, work function, surface properties, conductivity, and crystal phase, which can significantly enhance their sensing performance. Therefore, the incorporation of carbon atoms into the MOS framework endowed the materials with several merits: 1) more oxygen vacancies can be generated due to the substitution of atoms; 2) the surface polarity of the pore wall can be tuned to facilitate the adsorption towards target gas molecules; 3) specific crystal phase for selective interaction with target molecules can be preferentially formed.

#### 2.4.2 Nitrogen‐Doped Porous MOS

Apart from the carbon doping, the nitrogen doping in porous metal oxides can also remarkably enhance their gas sensing performance through unique mechanism. Abbasi and Sardroodi^[^ ^92^ ^]^ investigated the adsorption behavior of the pristine and N‐doped TiO_2_ anatase nanoparticles toward NO_2_ molecules by using the density functional theory (DFT) calculations. They found that the NO_2_ molecules was preferred to adsorb on the N‐doped TiO_2_ rather than the pristine ones, and the nitrogen atom in NO_2_ molecules could be preferentially adsorbed on the dangling oxygen and doped nitrogen atom sites of the TiO_2_ nanoparticles. The oxygen atom in NO_2_ molecules have a higher affinity towards the fivefold coordinated titanium atoms. In addition, the oxidation of NO_2_ to NO_3_ was more easily due to the breaking of the Ti‐O bonds between the nanoparticles and the NO_2_ molecules. Therefore, the N‐doped TiO_2_ nanoparticles showed superior sensing performance than the undoped ones.

**Figure 8:**
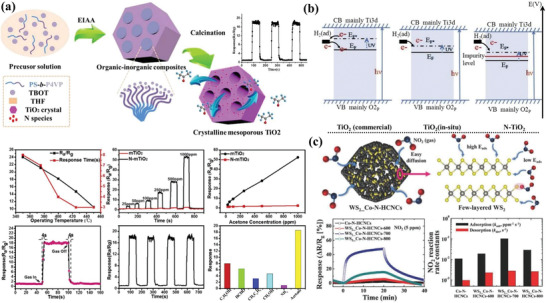
a) The synthesis route of the crystalline ordered mesoporous N‐doped TiO_2_ via EIAA strategy and their potential acetone sensing behaviors: including sensitivity, selectivity, and cycling stability. Adapted with permission.^[^ ^19^ ^]^ Copyright 2018, Elsevier. b) The electron transfer behavior over commercial TiO_2_, in situ TiO_2,_ and N‐doped TiO_2_ to adsorbing H_2_. Adapted with permission.^[^ ^93^ ^]^ Copyright 2019, Elsevier. c) The NO_2_ sensing mechanism of few‐layered Co, N‐doped C/WS_2_ nanocages, dynamic response behavior and calculated adsorption/desorption rate constant. Adapted with permission.^[^ ^95^ ^]^ Copyright 2018, Wiley.

In additional, Zhang et al.^[^ ^19^ ^]^ reported a simple EIAA method by using lab‐made amphiphilic diblock copolymer PS‐*b*‐P4VP as a structure directing agent and TBOT as a titanium source to synthesize the in situ N‐doped ordered mesoporous titania (**Figure** **8**a). XPS, UV–vis diffuse reflectance spectra, and Fourier‐transform infrared spectroscopy (FT‐IR) results showed that the nitrogen atoms were in situ incorporated into the lattice of TiO_2_, resulting in a smaller particle size and abundant oxygen vacancies, which was a benefit for the improvement of gas sensing performance. The obtained ordered mesoporous N‐TiO_2_ materials exhibited excellent gas sensing properties towards acetone, and the response was 11.4 times higher than the undoped TiO_2_‐based sensor, which was mainly due to the opened pore channels, large specific surface area, and more oxygen vacancy. The formation of impurity level induced by the N doping can also reduce the Fermi level of N‐doped TiO_2_, leading to more electrons transfer from the adsorbed target gas to N‐TiO_2_ (Figure 8b).^[^ ^93^ ^]^

The N‐doped sites also facilitated the adsorption of target gases due to their much active reactivity. For instance, the N‐doped porous ZnO was synthesized,^[^ ^94^ ^]^ and the unpaired electrons on the dopant nitrogen atoms were contributed to a negative‐differential resistance behavior of this device. In addition, the chemisorption evaluation of H_2_, O_2_ and CO_2_ molecules showed that the adsorption of H_2_ on the surface of materials might lead to the formation of an extended defect near the vicinity of N sites, and further enhanced their conductivity. However, the chemisorption of O_2_ and CO_2_ molecules can break the *π*‐bonds and pave the way to its two oxygen atoms to form two new chemical bonds O—N and O—Zn in case of O_2_ molecules. Hence, both O_2_ and CO_2_ molecules play a role of acceptor to drain charge from N‐sites and making it more depleted, resulting in the reduction of conductivity and an extension of the negative‐differential resistance range.

Instead of substitutional and interstitial doping N atoms into the lattice of semiconductors, the synergistic effect between N‐doped matrix and semiconductors are also contributed to the enhancement of gas sensing performance.^[^ ^95^ ^]^ Typically, MOFs templates are introduced to synthesize few‐layered WS_2_ nanoplates (a lateral dimension of ≈10 nm) confined in Co, N‐doped hollow carbon nanocages (WS_2_‐Co‐N‐HCNCs), for highly sensitive NO_2_ gas sensors (Figure 8c). During the pyrolysis, the Co and N co‐doped carbonized ZIF‐67 are formed, and the growth of WS_2_ is effectively suppressed, creating few‐layered WS_2_ nanoplates functionalized Co‐N‐HCNCs. On the one hand, the resistance of p‐type WS_2_ is decreased when NO_2_ molecules are adsorbed on the edge of 2H‐WS_2_. On the other hand, N‐doped hollow carbon matrix provided the easy diffusion path for NO_2_ molecules and electrically connected the WS_2_ network. Thus, the synergistic effect promoted the gas sensing performance of the obtained materials towards room temperature detection of NO_2_.

Therefore, the remarkable enhancement of their gas sensing performance derived from the N‐doped MOS materials can be attributed to the following aspects: 1) oxygen or target molecules can be preferentially adsorbed on the N‐doped sites due to the formation of surface vacancies; 2) the N‐doping can also form lower Fermi level, leading to the efficiency electrons transfer during the sensing process; 3) the synergistic effect between N‐doped matrix and MOS contributed to the enhancement of gas sensing performance.

#### 2.4.3 Silicon‐Doped Porous MOS

**Figure 9:**
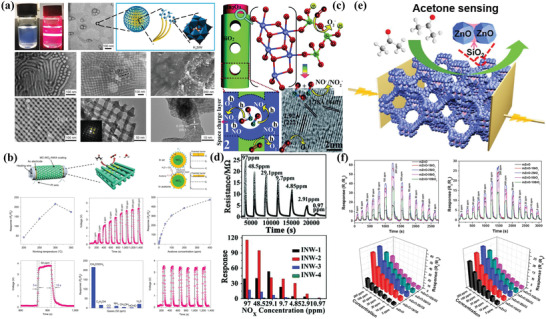
a) Coassembly of PEO‐*b*‐PS and H_4_SiW_12_O_40_ to synthesize 3D cross‐stacked Si‐doped WO_3_ nanowires and structural characterization. b) Gas sensing dynamic behavior and mechanism of Si‐doped *ε*‐WO_3_ nanowires‐based gas sensors toward acetone. Adapted with permission.^[^ ^88^ ^]^ Copyright 2020, Springer Nature. c) Structural and energy band models of the NO*_x_* sensing mechanism on the Si‐doped mesoporous In_2_O_3_ nanowires. d) Dynamic behavior at different NO*_x_* concentrations and gas sensitivities comparison of Si‐doped In_2_O_3_ with various In_2_O_3_ ratio. Adapted with permission.^[^ ^98^ ^]^ Copyright 2015, Royal Society of Chemistry. e) Acetone sensing behavior on amorphous SiO_2_‐cemented mesoporous ZnO. f) Response–recovery curves and concentration–response comparison toward acetone and ethanol, respectively. Adapted with permission.^[^ ^39^ ^]^ Copyright 2019, American Chemical Society.

Silicon‐doped semiconductors also exhibit superior gas sensing performance compared with the pristine one. It is worth noting that the *ε*‐WO_3_ is a metastable phase with a high selectivity to acetone, and the *ε*‐phase WO_3_ could be obtained by doping Si atoms into the framework.^[^ ^96^ ^]^ Typically, Deng's group^[^ ^88^, ^97^ ^]^ reported a general and flexible synthetic orthogonal assembly approach to controllably construct 3D cross‐stacked metal oxide nanowire arrays with well‐interconnected frameworks and uniform nanowire spacings (**Figure** **9**a). Using the BCP‐directed coassembly approach with various commercial or synthetic Keggin‐type POMs such as silicotungstic acid, silicomolybdic acid, phosphotungstic acid and phosphomolybdic acid, diverse multilayer‐crossed nanowire arrays of doped‐metal oxides with uniform nanowire thicknesses and spacings can be readily fabricated. Typically, the *ε*‐WO_3_ was elaborately investigated, and the local electronic and atomic structure of Si‐doped *ε*‐WO_3_ was measured by X‐ray absorption near‐edge spectroscopy and extended X‐ray absorption fine structure. Due to the unique structural properties of the 3D cross‐stacked metal oxide semiconducting nanowires, the obtained materials exhibited excellent acetone‐sensing performances with a high sensitivity (a limit of detection of 10.0 ppb) and high selectivity, fast response/recovery dynamics, and good stability (Figure 9b). Therefore, Righettoni et al.^[^ ^87^ ^]^ investigated the effect of nontoxic Si doping on the *ε*‐phase content and crystal structure on the acetone sensing performance, and the results showed that the sensor baseline (resistance) increased steeply by 4 orders of magnitude with the increasing of Si content from 0 to 40 mol%. When the doping amount was 10% in the framework, the materials exhibited the best sensing performance towards acetone. In addition, the *ε*‐phase WO_3_ can be maintained at 500 °C without grain growth, and the obtained materials show ultralow detection limit toward 20 ppb acetone with high selectivity. This work paved a new way to optimize the sensing performance of MOS‐based gas sensors. Later on, many research groups carried out further investigation on the sensing performance Si‐doped materials.

Moreover, the silica doping amount can also affect the sensing properties. Typically, the Si‐doping in MoO*_x_* nanoparticles inhibited the sintering and crystal growth during the high‐temperature treatment.^[^ ^89^ ^]^ XRD results showed that the incorporation of Si^4+^ into the orthorhombic MoO_3_ lattice could lead to the lattice parameter expansion in the *b*‐axis from 13.858 to 13.871 Å and in the c‐axis from 3.697 to 3.701 Å, indicating the formation of the interstitial solid solutions during the flame spray pyrolysis process. When the silica doping amount was above 1.5%, the excess Si was not incorporated into the orthorhombic MoO_3_ lattice and formed domains of SiO_2_. In addition, the Si‐doped MoO_3_ sensors were performed to detect the NH_3_ molecules, and the materials exhibited high selectivity toward other breath‐relevant gases (such as acetone, NO, CO) compared with pure MoO_3_. When the silica doping amount was beyond 1.5%, the segregated SiO_2_ could narrow the conduction channel locally, resulting in the increase of film resistance and improved NH_3_ sensitivity.

In addition, 1D highly crystalline mesoporous In_2_O_3_ nanowires (INWs) coated by an amorphous silica (HCMIAS) ultrathin surface layer were successfully fabricated using SBA‐16 as a hard template.^[^ ^98^ ^]^ The silica doping in the In_2_O_3_ nanowires endowed the materials with high surface areas and much thicker space‐charge layer, which promised the effective interactions between target gases and abundant active sites (Figure 9c). In addition, the silica‐modified surface is also favored for the effective adsorption of NO*_x_* species, resulting in the enhancement of their gas sensing performance. Compared with the pure In_2_O_3_ nanowires, the Si‐doped In_2_O_3_ nanowires exhibited ultrahigh response and selectivity with low detection limit of 0.97 ppm toward NO*_x_* at room temperature (Figure 9d). Interestingly, the switchable sensing selectivity of ZnO can be achieved by using Si‐doping strategy. In this regard, Zhou et al.^[^ ^39^ ^]^ also reported a cementing mesoporous ZnO with silica for controllable and switchable gas sensing selectivity (Figure 9e). Particularly, novel silica‐cemented mesoporous ZnO materials with different contents of silica, high surface areas, and well‐interconnected pores (29 nm) are synthesized through the solvent evaporation‐induced coassembly (EICA) approach. For comparison, the pure mesoporous ZnO showed better selectivity towards ethanol. When the silica doping amount was about 2 wt%, the obtained cementing mesoporous ZnO with silica exhibited superior selectivity towards acetone rather than ethanol (Figure 9f). Both gas chromatograph‐mass spectrum (GC‐MS) analysis and intelligent gravimetric analyzer (IGA) measurement indicated that the silica doping in mesoporous ZnO could modify the surface properties, and it exhibited enhanced adsorption ability towards the polar acetone molecules. Therefore, the tunable gas sensing selectivity can be achieved by doping different amounts of silica.

As mentioned above, the silica‐doping strategy can modify the surface property of semiconductors, which promises the switchable gas sensing selectivity. In addition, the silica‐doping can change the crystalline phase of the semiconductor and inhibit the growth of grain size, which also facilitates the improvement of gas sensing performance.

#### 2.4.4 Sulfur‐Doped Porous MOS

**Figure 10:**
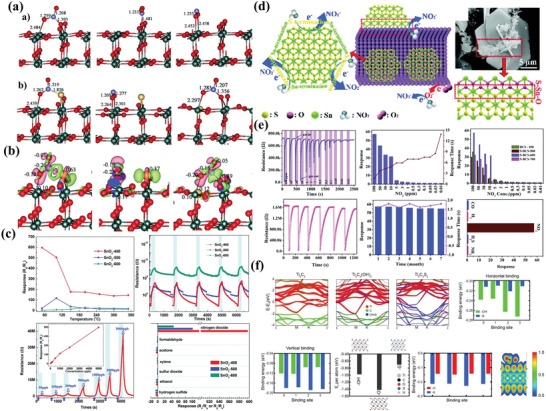
a) The optimized adsorption configurations for NO_2_ adsorption on the surface of a) SnO_2_(110) and b) S‐doped SnO_2_(110). b) The corresponding charge density differences for NO_2_ adsorption on the surface of S‐doped SnO_2_(110). Gray‐Sn atom; red‐O atom; yellow‐S atom and blue‐N atom. c) NO_2_ sensing behavior on S‐doped SnO_2_ treated by various sintering temperatures. Adapted with permission.^[^ ^91^ ^]^ Copyright 2018, Royal Society of Chemistry. d) NO_2_ sensing mechanism on S‐doped SnO_2_ and potential electron flow direction. e) Dynamic sensing behavior and response testing of NO_2_ gas on fabricated S‐doped SnO_2_‐based sensors. Adapted with permission.^[^ ^90^ ^]^ Copyright 2020, Royal Society of Chemistry. f) Energy band structure, formation energy, horizontal binding energy, and charge distribution variation for S‐doped Ti_3_C_2_T*_x_* MXene toward adsorbed VOCs molecule. Adapted with permission.^[^ ^99^ ^]^ Copyright 2020, American Chemical Society.

Sulfur doping is also an intelligence route to enhance the gas sensing performance of semiconductors. Particularly, the formed sulfide or S‐doped composites facilitates the charge transfer between oxide and sulfide. For example, Xu et al.^[^ ^91^ ^]^ investigated the sensing performance of flower‐like mesoporous sulfur‐doped SnO_2_ materials towards NO_2_ molecules at low working temperature (50 °C). It also showed that the sulfur doping in SnO_2_ could modify their surface properties with high chemical reactivity, and the gas sensing performance was closely related to the content of sulfur doping. Both in situ Raman and DFT results showed that the sulfur‐doped SnO_2_ materials exhibited super catalytic activity and improved absorptivity (**Figure** **10**a–c). Notably, Li et al.^[^ ^90^ ^]^ have also synthesized the mesoporous SnO_2_ by using the biomass carbon as the template. Then S‐doped biomorphic SnO_2_ was prepared by the CVD method, and SnS_2_ was formed and could be regarded as the active chemical reaction sites to interact with the target NO_2_ molecules, inducing the enhanced gas sensing performance (Figure 10d). It demonstrated that the number of S—Sn—O chemical bonds was highly dependent with the gas sensing performance, and the sulfur doping could also reduce the interface state density and increase the carrier density. It can facilitate the formation of surface oxygen vacancies, resulting in the improvement of the gas sensing performance (Figure 10e). In addition, Shuvo et al.^[^ ^99^ ^]^ reported that S‐doping of Ti_3_C_2_T*_x_* MXene materials for the detection of VOCs compared with the undoped counterparts. Upon doping sulfur into the framework, the interlayer spacing of the MXene nanoflakes increased substantially, and the sensing performance towards toluene was three to four folds increased compared with the undoped counterparts. DFT results indicated that the binding energy of toluene to the S‐functionalized MXenes increased substantially, and the toluene molecules were favored for adsorbing around the S atoms, resulting in the enhancement of the gas response (Figure 10f). Thus, it can be seen that the sulfur doping can mainly modify the surface properties of semiconductors with S‐functionalized species, which facilitates the adsorption and catalytic reaction of the target gas molecules.

## 3 Conclusion and Perspective

Nowadays, with the globally increasing demands for high‐performance sensors, new opportunities and challenges for MOS‐based gas sensors have emerged. Particularly, porous materials with larger specific surface areas can provide numerous active sites for the adsorption of target gas molecules and facilitate the surface catalytic reaction at the interface of porous MOS. In addition, the well‐connected pore channels and large pore size can also enhance the diffusion of oxygen or target gases inside the framework. In this review, we systematically illustrated the synthesis strategy of mesoporous MOS, including templating techniques (soft‐ and hard‐template), mechanochemical nanocasting method and other advanced strategies. In addition, apart from the design of gas‐diffusion‐favored structure, introducing additives in semiconducting metal oxides is the most common way to tune the sensitivity, selectivity, and stability of chemoresitsive gas sensors. Therefore, several strategies such as the construction of heterojunctions, modification with noble metals and incorporation with alternative heteroatoms doping have been elaborately discussed. The related chemical and electrical sensitization mechanisms of the introduced additives as well as the role of different structures have been rationally illustrated. Based on these strategies, it is believed that numerous high‐performance porous MOS gas sensing material system toward different target gases can be achieved in the future. Despite the obvious progress, there are still scientific and practical issues that need to be resolved.

1) Notably, various porous MOS sensitive materials and sensing mechanisms have been reported, however, the poor selectivity and stability of the sensors are still great issues in practical applications. In addition, the environment humidity can remarkably affect the sensing sensitivity of porous MOS owing to the complicated surface adsorption‐desorption and catalysis process. In this case, the sensing performance can deteriorate in high humidity conditions (e.g., the exhaled gas detection), which is unfavorable for reliable detection of target gas in different humidity. In addition, a high response with low interfering sensitivity is important for accurate detection, and the improvement of selectivity can avoid false warning in practical application. In this regard, the surface modification can be performed to achieve hydrophobic surface properties to prevent water adsorption and avoid the influence of humidity, and the precise design of surface electronic structure and surface oxygen species are highly desired for the enhancement of their selectivity and stability.
2) Importantly, researchers should pay more attention to the catalytic effect of metal oxide based sensors in the future. The transition metal elements possess unfilled valence d orbitals, and the MOS‐based materials exhibited unexpected catalytic activity. In addition, the sensing mechanisms of gas sensors involved the gas adsorption, surface activation, surface reaction, and desorption, which is highly relevant to the surface catalytic reaction process. Therefore, the surface properties such as the surface acidity/basicity, the oxygen vacancies and the active oxygen species can greatly influence the sensing performance and sensing selectivity. However, in most cases, the basic sensing mechanisms are based on the electron depletion layer (EDL) theory, the surface oxygen adsorption theory, and the gas‐solid catalysis process. Therefore, the catalytic effect of MOS‐based materials should be considered in addition to the surface adsorption and the EDL process. In addition, the sensing process involves complicated surface adsorption‐desorption, band bending induced chemiresistance variation, and the catalytic reaction mechanism of target gas over the porous MOS should be systematically investigated. Hence, advanced characterizations such as in situ solid nuclear magnetic resonance (NMR), in situ FT‐IR, in situ Raman, in situ extended X‐ray absorption fine structure (EXAFS) analysis which have been widely employed in the catalysis can be performed to reveal the fine structure changes during gas sensing process. Overall, it is still necessary to explore new mesoporous MOS materials (e.g., multicomponent materials and heterojunction materials) and flexible sensing devices to meet the requirements in practical applications. Mesoporous MOS materials with single atom‐doped frameworks, 2D structure, and 1D nanowire heterojunction hold great potential for the development of high‐performance miniaturized sensors. Based on nanodevices with integrated mesoporous MOS sensing layer, it is possible to carry out in situ study and further disclose the intrinsic component–structure–function relationship, which can promote the development of gas sensing technology. Additionally, the combination of theoretical simulation and experiment should be further developed to guide the mechanism exploration and materials design.
3) With the increasing requirement of electronic device miniaturization, the low working temperature with low power consumption is becoming the development trend in the future, and thus high‐performance MEMS sensors with integrated sensitive layers are a promising field. Nevertheless, novel fabrication technologies should be explored to integrate mesoporous MOS materials with MEMS chips to form reliable and stable gas sensing nanodevices. Additionally, the manufacturing technologies and industrialization platform technologies for MOS‐based gas‐sensitive film are facing three challenges: poor selectivity, poor stability, and low consistency in device quality. Thus, the design of high‐performance MOS‐based sensing materials is important to fabricate the MEMS gas sensing chip.

In summary, this critical review is expected to provide a comprehensive understanding of the gas‐sensing enhancement strategies for mesoporous MOS‐based sensors. With the development of sensing technology and the advance in designing and synthesizing functional materials, we believe that rational functionalization of mesoporous MOS materials has great potential to create high‐performance sensing layers on various sensing substrates such as MEMS chips and wearable flexible nanodevices, bringing breakthroughs in developing miniaturized, integrated, portable, and intelligent gas sensors in the future.

## Conflict of Interest

The authors declare no conflict of interest.
